# A New Plant Indicator (*Artemisia lavandulaefolia* DC.) of Mercury in Soil Developed by Fourier-Transform Near-Infrared Spectroscopy Coupled with Least Squares Support Vector Machine

**DOI:** 10.1155/2019/3240126

**Published:** 2019-09-09

**Authors:** Lu Xu, Qiong Shi, Bang-Cheng Tang, Shunping Xie

**Affiliations:** ^1^College of Material and Chemical Engineering, Tongren University, Tongren 554300, Guizhou, China; ^2^The Modernization Engineering Technology Research Center of Ethnic Minority Medicine of Hubei Province, College of Pharmacy, South-Central University for Nationalities, Wuhan 430074, China; ^3^Technology Center, China Tobacco Guizhou Industrial Co., Ltd., Guiyang 550009, Guizhou, China

## Abstract

A rapid indicator of mercury in soil using a plant (*Artemisia lavandulaefolia* DC., ALDC) commonly distributed in mercury mining area was established by fusion of Fourier-transform near-infrared (FT-NIR) spectroscopy coupled with least squares support vector machine (LS-SVM). The representative samples of ALDC (stem and leaf) were gathered from the surrounding and distant areas of the mercury mines. As a reference method, the total mercury contents in soil and ALDC samples were determined by a direct mercury analyzer incorporating high-temperature decomposition, catalytic adsorption for impurity removal, amalgamation capture, and atomic absorption spectrometry (AAS). Based on the FT-NIR data of ALDC samples, LS-SVM models were established to distinguish mercury-contaminated and ordinary soil. The results of reference analysis showed that the mercury level of the areas surrounding mercury mines (0–3 kilometers, 7.52–88.59 mg/kg) was significantly higher than that of the areas distant from mercury mines (>5 kilometers, 0–0.75 mg/kg). The LS-SVM classification model of ALDC samples was established based on the original spectra, smoothed spectra, second-derivative (D2) spectra, and standard normal transformation (SNV) spectra, respectively. The prediction accuracy of D2-LS-SVM was the highest (0.950). FT-NIR combined with LS-SVM modeling can quickly and accurately identify the contaminated ALDC. Compared with traditional methods which rely on naked eye observation of plants, this method is objective and more sensitive and applicable.

## 1. Introduction

Excessive mercury is one of the major heavy metal pollutants in the environment, which can cause great toxicity to human body [1–3]. A series of environmental pollution incidents have aroused great attention to mercury pollution in the world [4]. In recent years, the study of mercury pollution in soil has become one of the hotspots of environmental protection [5]. Mercury exists in soils in different forms, such as Hg^0^, Hg_2_^2+^, Hg^2+^, and organic mercury. There is also a complex interaction between different forms of mercury under specific environmental conditions; for example, mercury can be converted into highly toxic methylmercury in the soil [6]. Because of the hysteresis, easy migration, and high toxicity of mercury pollution in soil, the analysis and detection technology of mercury in soil is of great significance to soil monitoring and the effective control of mercury pollution. Therefore, in recent years, the development of trace mercury extraction, enrichment, and detection methods [7] has become one of the hot research fields of analytical chemistry.

At present, most of the analytical methods for total mercury in soil involve cumbersome, time-consuming, and reagent-consuming sample pretreatment and extraction methods, such as wet digestion, dry ashing, microwave digestion, and ultrasound-assisted extraction. [8]. The quantitative detection methods include spectrophotometry [9, 10], atomic spectrometry [11], chromatography [12], mass spectrometry [13], neutron activation analysis [14], and so on [15–17]. Tongren City (Guizhou Province, China) has many large-scale mercury deposits. Mercury deposits play an important role in the local traditional industry, but they also pose a major threat to the ecological environment. The traditional quantitative analysis method of mercury needs more complex experimental operation skills and analytical instruments. The number of samples to be routine analyzed is large, and the cost of analysis and testing is high, so it cannot be fully popularized in economically underdeveloped areas.

Indicator plants [18, 19] are sensitive to pollutants and show obvious morphological changes in the presence of pollutants, but the distribution of indicator plants is greatly affected by geographical and climatic factors. In the case of long-term soil contamination, the morphological changes of most local species are often not obvious due to tolerance. Therefore, the application of traditional methods of plant indicators by naked eye observation is largely limited. Fourier-transform near-infrared (FT-NIR) spectroscopy, as a fast detection method, has the advantages of convenient sample preparation, rapid analysis, and simultaneous characterization of mixtures [20, 21]. Combined with chemometrics, FT-NIR has been widely used in the classification of various samples [22–24]. In order to screen the mercury contamination in local soil rapidly and effectively, in this work, *Artemisia lavandulaefolia* DC. (ALDC), a local plant commonly distributed in mercury mining areas, was investigated as a mercury indicator. FT-NIR spectroscopy was used to characterize the chemical composition changes caused by mercury in ALDC. Finally, chemometrics methods were applied to identify its mercury content level rapidly by developing classification models.

## 2. Materials and Methods

### 2.1. Collection of ALDC Samples and FT-NIR Spectrometry

ALDC samples were collected including stem and leaves at the top of the plants with a length of about 20 cm. The mercury-contaminated group was collected within 3 km surrounding mercury mines (*n*_1_ = 120), and the regular group was gathered from areas farther from mercury mines (>5 km and *n*_2_ = 120). All ALDC samples are washed and stored in a cool, dry, and ventilated place to avoid direct sunlight to remove moisture. Each sample was crushed by a crusher and then passed through a 200-mesh sieve. Samples powders were stored with integrated packaging. Before FT-NIR analysis and Hg reference analysis, each sample was dried by an ultraviolet lamp for 10 minutes. An Antaris II Fourier-transform near-infrared spectrometer (Thermo Electron Co., USA) was used to analyze the compacted powder in a quartz sample cup under reflection mode. A PbS detector was used to record the spectrum. The measured spectral interval was 4000–10000 cm^−1^. Each sample is measured three times, and the number of scans per measurement was 32. The resolution of the instrument is 8 cm^−1^.

### 2.2. Reference Analysis of Mercury

Reference analysis of mercury was performed using a DMA-80 direct mercury analyzer (Milestone, Italy). Mercury standard reserve solution (100 mg/L) was purchased from the standard sample research center of China Ministry of Environmental Protection. Mercury standard solutions (10.0 mg/L and 1.0 mg/L) were obtained by diluting the standard reserve solution with 1% (w/w) nitric acid and deionized water. The purity of oxygen was over 99.99% (v/v).

The working conditions of the DMA-80 direct mercury analyzer were as follows: low pressure mercury lamp was used as light source; the wavelength was kept at 253.7 nm; drying temperature was kept at 200°C for 60 s; decomposition temperature was kept at 650°C for 90 s; the oxygen pressure was 60 psi; and the detector was silicon ultraviolet photoelectricity. For each ALDC (naturally dried in the sun) or soil sample, the weight was kept at 100 mg and accurately weighted. The standard curve was developed by using the following series of standard solutions with 0, 0.2, 0.4, 0.6, 0.8, and 1.0 mg/L.

### 2.3. Chemometrics Analysis

Data preprocessing and classification modeling were both computed on MATLAB 7.0.1 (MathWorks, USA). In order to obtain representative training set and test set, the Kennard–Stone (K-S) algorithm [25] was used to divide the original spectral data. In the K-S method, first, the two samples with the greatest Euclidean distance are selected as training objects; and then the two samples with the greatest distance from the remaining samples are selected and put into the test set. The above process is repeated until one has obtained enough test objects and all the remaining objects are put into the training set. The training and test sets obtained by the K-S algorithm can cover a wide range of spectral data. The code for the K-S algorithm comes from the free TOMCAT toolbox [26]. Least square support vector machine (LS-SVM) [27] is used for classification modeling and prediction. The kernel width and normalized parameters of the Gauss function in LS-SVM model were estimated by the cross-validation method. LS-SVM was performed using LS-SVMlab 1.8 toolbox [28]. The second-order derivative (D2) [29] and standard normal transformation (SNV) [30] spectra were calculated using self-compiled MATLAB code.

## 3. Results and Discussion

### 3.1. Mercury Content in Samples

According to previous research experience, there is little difference in mercury content between plants near the same sampling location (within the range of 200 meters), and considering that the rapid spectral identification method is qualitative, the mercury content of 10 batches of samples near mercury mines and 11 batches of samples far away from mercury mines was determined in this paper as a reference for the rapid spectral identification method. According to the standard curve, the linear range of the method is 0–1.0 mg/L, and the linear correlation coefficient is greater than 0.999. According to the definition of IUPAC, the detection limit was calculated to be 0.2 *μ*g/L by 3-fold standard deviation of 11 repeated measurements of blank solution under the same conditions as the sample solution. Quantitative analysis showed that there were significant differences in mercury content between the two groups near and far away from mercury deposits, ranging from 0 to 0.75 mg/kg and 7.52–88.59 mg/kg, respectively.

### 3.2. FT-NIR Spectra and Data Preprocessing

**Figure 1:**
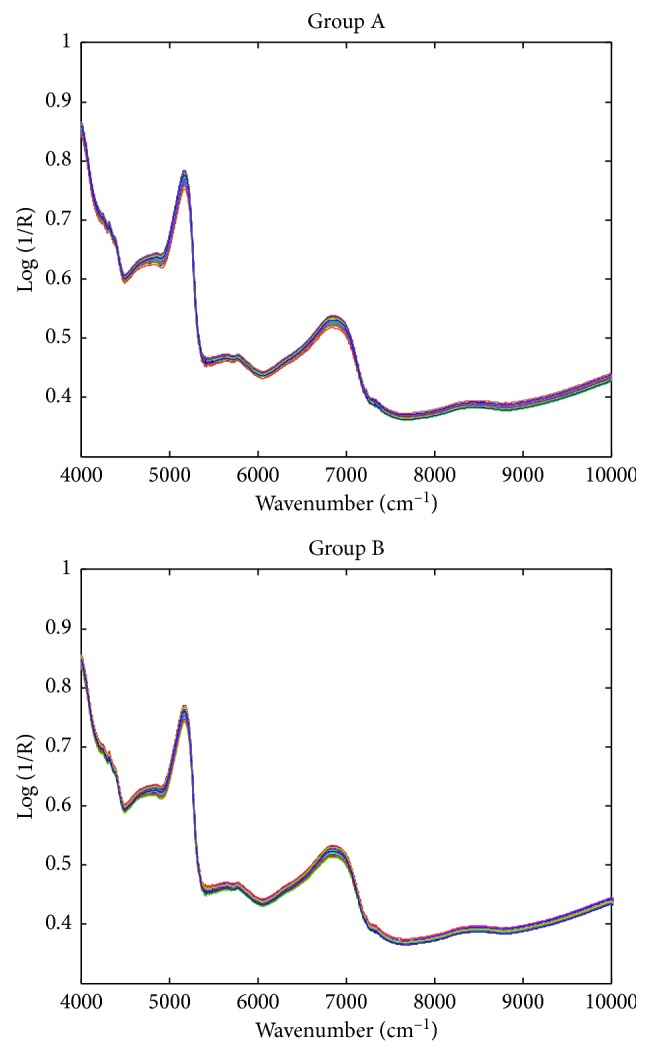
Raw FT-NIR spectra of regular (group A) and mercury-contaminated (group B) ALDC samples.

**Figure 2:**
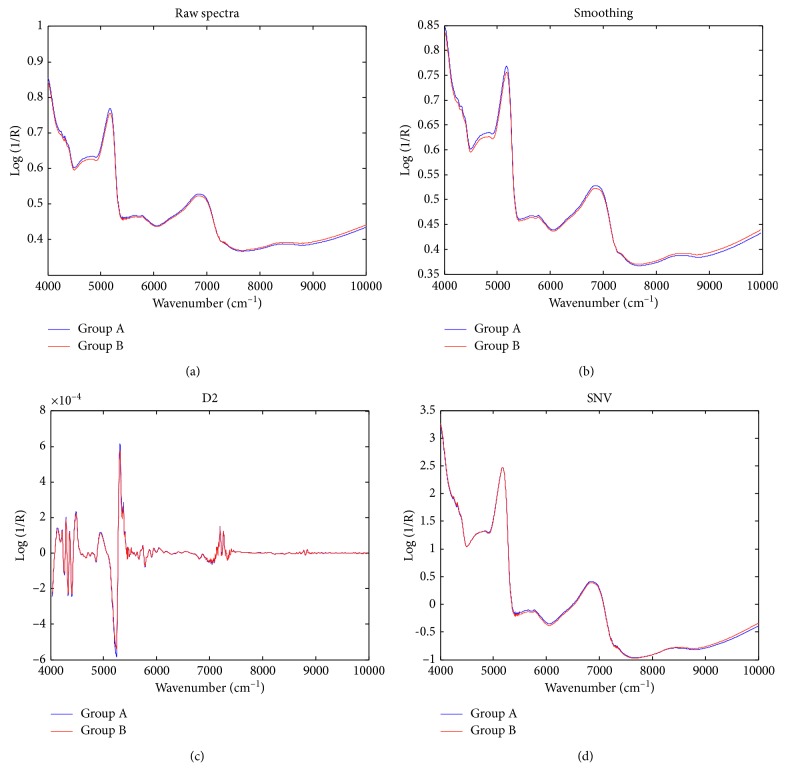
Raw and preprocessed averages of FT-NIR spectra of regular (group A) and mercury-contaminated (group B) ALDC samples.

Figure 1 shows the original FT-NIR spectra of the two groups of samples. According to the original spectra, although the relative intensity of some peaks may be different, the absorption bands are basically similar. It is difficult to distinguish them by naked eyes, so it is necessary to use chemometrics to model and classify them. In order to further eliminate the scattering effect of sample powders and the possible baseline drift, D2 and SNV spectra were also calculated. As shown in Figure 2, the D2 spectrum can eliminate most of the baseline effects. Near 7250 cm^−1^, the group A samples (mercury-polluted) have more significant changes in spectral intensity, so the D2 spectrum has stronger peaks nearby. After SNV transformation, the intraclass spectral differences between the two groups were reduced, but the differences between the two groups were still not obvious. Chemometrics should be used for classification modeling.

### 3.3. Classification Modeling

**Figure 3:**
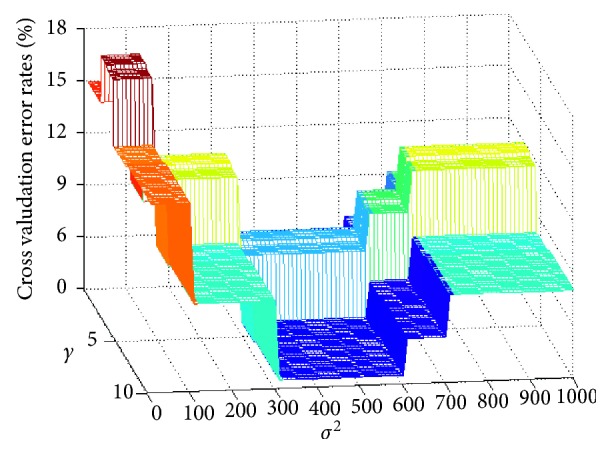
Optimization of LS-SVM model parameters (group A, 1–40; group B, 41–80). (a) Smoothing. (b) D2. (c) SNV.

**Figure 4:**
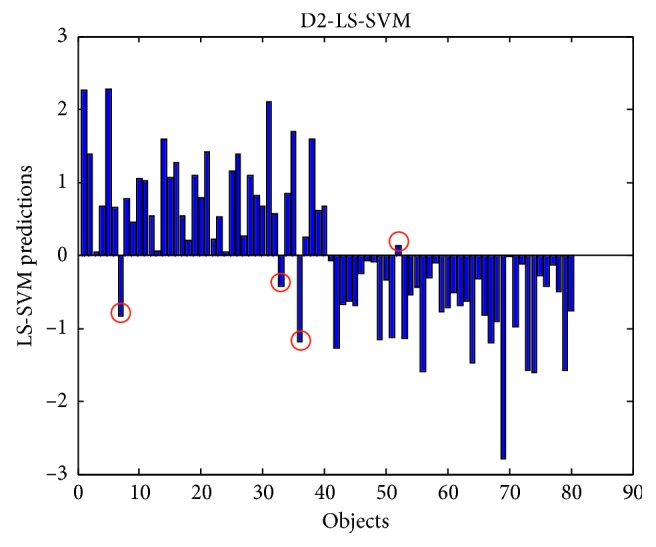
The predictions of regular (group A) and mercury-contaminated (group B) ALDC samples by D2-LS-SVM.

Considering the difference of sample distribution between the two groups, the K-S algorithm was used to divide the A and B group into 80 training samples and 40 test samples separately. Therefore, the final training set contained 160 (80 + 80) samples and 80 (40 + 40) samples, respectively. The LS-SVM model was constructed based on original spectrum, smoothed spectrum, D2 spectrum, and SNV spectrum. In this paper, LS-SVM used the usual Gaussian kernel function as the nonlinear transformation, so it is necessary to optimize two parameters simultaneously, namely, the kernel width (*σ*) of the Gaussian kernel function and the normalized parameter (*γ*). The simplex method was used to optimize the classification error rate of 10-fold cross verification.  Table 1 lists the model parameters and prediction results using different data preprocessing methods. In order to demonstrate the optimization of LS-SVM parameters, Figure 3 shows the error rates of cross validation obtained using different pairs of *σ*^2^ and *γ*. As shown in Figure 3, there is a platform where the error rates reach the lowest value. For a stable and accurate model, the optimal pairs of *σ*^2^ and γ were selected to be *σ*^2^ = 450 and *γ* = 7.5, which are located in the center of the platform. With the optimized parameters, the LS-SVM model was built to predict the classes of test samples. The error rates of cross validation of original spectrum-LS-SVM, smoothing-LS-SVM, D2-LS-SVM, and SNV-LS-SVM were 0.075, 0.069, 0.038, and 0.044, respectively. The prediction results of D2-LS-SVM are shown in Figure 4, indicating 4 of the 80 test objects were wrongly classified. The prediction results of 80 prediction objects also showed that D2 and SNV can eliminate some scattering effects and baseline drift, and the classification accuracy reached 0.950 and 0.925, respectively.

## 4. Conclusions

In this paper, the DMA-80 direct mercury analyzer was used as a reference analysis method to study the stem and leaf samples of ALDC from the areas surrounding and far away from the mercury mine. The results showed that the total mercury contents of the two samples were significantly different. Combining FT-NIR spectroscopy and pattern recognition method, the classification model of the two groups of ALDC samples was established and the classification accuracy was satisfactory. The comparison of different data preprocessing methods showed that SNV and D2 can eliminate the effects of partial scattering and baseline drift, highlight the spectral differences between the two groups, and obtain the improved classification accuracy of 0.950 and 0.925, respectively. FT-NIR combined with the pattern recognition method can quickly and accurately identify mercury-contaminated ALDC samples. Compared with the traditional method of naked eye observation, this method is more objective and can identify the changes of plant components caused by pollution more effectively.

## Figures and Tables

**Table 1:** Classification of regular and mercury-contaminated ALDC samples by FT-NIR and LS-SVM.

Preprocessing	Parameters (*σ*^2^, *γ*)	Error rates of cross validation	Prediction accuracy
Raw data LS-SVM	(840, 2.5)	0.075	0.912
Smoothing-LS-SVM	(800, 4.0)	0.069	0.875
D2-LS-SVM	(450, 7.5)	0.038	0.950
SNV-LS-SVM	(720, 4.5)	0.044	0.925

## Data Availability

The data used to support this study are available upon request by interested readers.

## References

[1] Aschner M., Onishchenko N., Ceccatelli S. (2010). Toxicology of alkylmercury compounds. *Organometallics in Environment and Toxicology: Metal Ions in Life Sciences*.

[2] Wu D., Chen L., Lee W., Ko G., Yin J., Yoon J. (2018). Recent progress in the development of organic dye based near-infrared fluorescence probes for metal ions. *Coordination Chemistry Reviews*.

[3] Meng A., Xu Q., Zhao K., Li Z., Liang J., Li Q. (2018). A highly selective and sensitive “on-off-on” fluorescent probe for detecting Hg(II) based on Au/N-doped carbon quantum dots. *Sensors and Actuators B: Chemical*.

[4] Rasheed T., Nabeel F., Li C., Bilal M. (2019). Rhodamine-assisted fluorescent strategy for the sensitive and selective in-field mapping of environmental pollutant Hg(II) with potential bioimaging. *Journal of Luminescence*.

[5] Han C., Wang H., Xie F., Wang W., Zhang T. A., Dreisinger D. (2019). Feasibility study on the use of thiosulfate to remediate mercury-contaminated soil. *Environmental Technology*.

[6] Yardim M. F., Budinova T., Ekinci E., Petrov N., Razvigorova M., Minkova V. (2003). Removal of mercury (II) from aqueous solution by activated carbon obtained from furfural. *Chemosphere*.

[7] Zheng H., Hong J., Luo X. (2019). Combination of sequential cloud point extraction and hydride generation atomic fluorescence spectrometry for preconcentration and determination of inorganic and methyl mercury in water samples. *Microchemical Journal*.

[8] Rodolfo F. M., Rucandio I. (2013). A simplified method for determination of organic mercury in soils. *Analytical Methods*.

[9] Wang S., Song X., Hu J. (2019). Direct speciation analysis of organic mercury in fish and kelp by on-line complexation and stacking using capillary electrophoresis. *Food Chemistry*.

[10] Chen B., Ma J., Yang T., Chen L., Gao P. F., Huang C. Z. (2017). A portable RGB sensing gadget for sensitive detection of Hg2+ using cysteamine-capped QDs as fluorescence probe. *Biosensors and Bioelectronics*.

[11] Lisboa M. T., Clasen C. D., Oreste E. Q., Ribeiro A. S., Vieira M. A. (2015). Comparison between vapor generation methods coupled to atomic absorption spectrometry for determination of Hg in glycerin samples. *Energy & Fuels*.

[12] Cheng H.-Y., Zhang W.-W., Wang Y.-C. (2018). Graphene oxide as a stationary phase for speciation of inorganic and organic species of mercury, arsenic and selenium using HPLC with ICP-MS detection. *Microchimica Acta*.

[13] Rahman G. M. M., Wolle M. M., Fahrenholz T., Kingston H. M. S., Pamuku M. (2014). Measurement of mercury species in whole blood using speciated isotope dilution methodology integrated with microwave-enhanced solubilization and spike equilibration, headspace-solid-phase microextraction, and GC-ICP-MS analysis. *Analytical Chemistry*.

[14] Lo J. M., Wei J. C., Yeh S. J. (1978). Determination of mercury in human urine by neutron activation analysis, with lead diethyldithiocarbamate as a preconcentration agent. *Analytica Chimica Acta*.

[15] Roditi E., Tsetsoni M., Kokkinos C., Economou A. (2019). Integrated on-chip sensor with sputtered Ag-Au-Au electrodes for the voltammetric determination of trace Hg(II). *Sensors and Actuators B: Chemical*.

[16] Li L., Zhang L.-P., Zhao Y. (2018). Colorimetric detection of Hg(II) by measurement the color alterations from the “before” and “after” RGB images of etched triangular silver nanoplates. *Microchimica Acta*.

[17] Yang H., Wang S., Wang Z. (2019). Task-specific ionic liquid-enabled mercury sensor for sensitive detection of total mercury in food digestion solution. *Sensors and Actuators B: Chemical*.

[18] Zahir F., Rizwi S. J., Haq S. K., Khan R. H. (2005). Low dose mercury toxicity and human health. *Environmental Toxicology and Pharmacology*.

[19] Grag P., Chandra P. (1994). The duckweed Wolffia globosa as an indicator of heavy metal pollution: sensitivity to Cr and Cd. *Environmental Monitoring and Assessment*.

[20] Qu J.-H., Liu D., Cheng J.-H. (2015). Applications of near-infrared spectroscopy in food safety evaluation and control: a review of recent research advances. *Critical Reviews in Food Science and Nutrition*.

[21] Costa F. R. C., Lang C., Almeida D. R. A., Castilho C. V., Poorter L. (2018). Near-infrared spectrometry allows fast and extensive predictions of functional traits from dry leaves and branches. *Ecological Applications*.

[22] Niu X., Zhao Z., Jia K., Li X. (2012). A feasibility study on quantitative analysis of glucose and fructose in lotus root powder by FT-NIR spectroscopy and chemometrics. *Food Chemistry*.

[23] Chitra J., Ghosh M., Mishra H. N. (2017). Rapid quantification of cholesterol in dairy powders using Fourier transform near infrared spectroscopy and chemometrics. *Food Control*.

[24] Xu L., Yan S.-M., Cai C.-B., Yu X.-P. (2013). Untargeted detection and quantitative analysis of poplar balata (PB) in Chinese propolis by FT-NIR spectroscopy and chemometrics. *Food Chemistry*.

[25] Kennard R. W., Stone L. A. (1969). Computer aided design of experiments. *Technometrics*.

[26] Daszykowski M., Serneels S., Kaczmarek K., Van Espen P., Croux C., Walczak B. (2007). TOMCAT: a MATLAB toolbox for multivariate calibration techniques. *Chemometrics and Intelligent Laboratory Systems*.

[27] Suykens J. A. K., Vandewalle J. (1999). Least squares support vector machine classifiers. *Neural Processing Letters*.

[28] De Brabanter K., Karsmakers P., Ojeda F. *Least Square Support Vector Machine MATLAB Tool Box*.

[29] Savitzky A., Golay M. J. E. (1964). Smoothing and differentiation of data by simplified least squares procedures. *Analytical Chemistry*.

[30] Barnes R. J., Dhanoa M. S., Lister S. J. (1989). Standard normal variate transformation and de-trending of near-infrared diffuse reflectance spectra. *Applied Spectroscopy*.

